# Supplementary material for: Bat teeth illuminate the diversification of mammalian tooth classes
Source: Nat Commun. 2023 Aug 22;14:4687. doi: 10.1038/s41467-023-40158-4 (PMC10444822; doi:10.1038/s41467-023-40158-4)
Supplement: Supplementary file 1 — Supplementary Information File [file 41467_2023_40158_MOESM1_ESM.pdf]

Supplementary information

Supplementary Fig 1-6
Supplementary Table 1
Supplementary Note 1

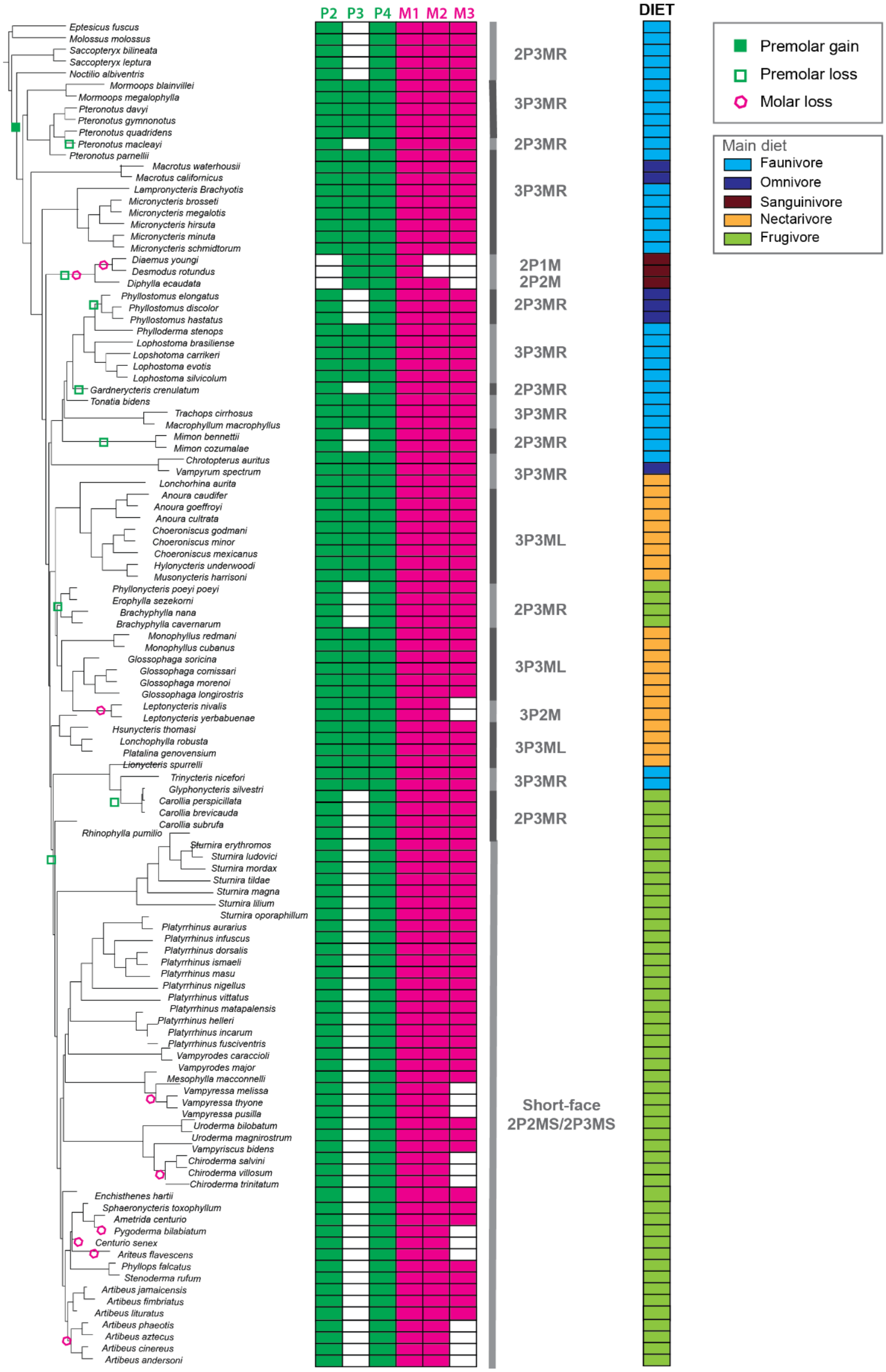

Supplementary Figure 1: Tooth number, jaw length and diet of the different bats

Phylogeny of all the species used in our dataset, their dental formula and the seven initial groups based on jaw length and tooth number (see Supplementary Tables 2 and 4). The morphogroup "Long jaw" contains the bats 3P3ML, the morphogroup "Regular jaw" contains the bats 3P3MR, the morphogroup "Intermediate jaw" contains the bats 2P3MR and the morphogroup "Short jaw" contains the bats 2P3MS and 2P2MS.For each species, the main diet is indicated. The events of premolar and molar gains and losses are also indicated on the tree.

Data sets can be found in Supplementary Data 1 and 2 and Source Data File 1.

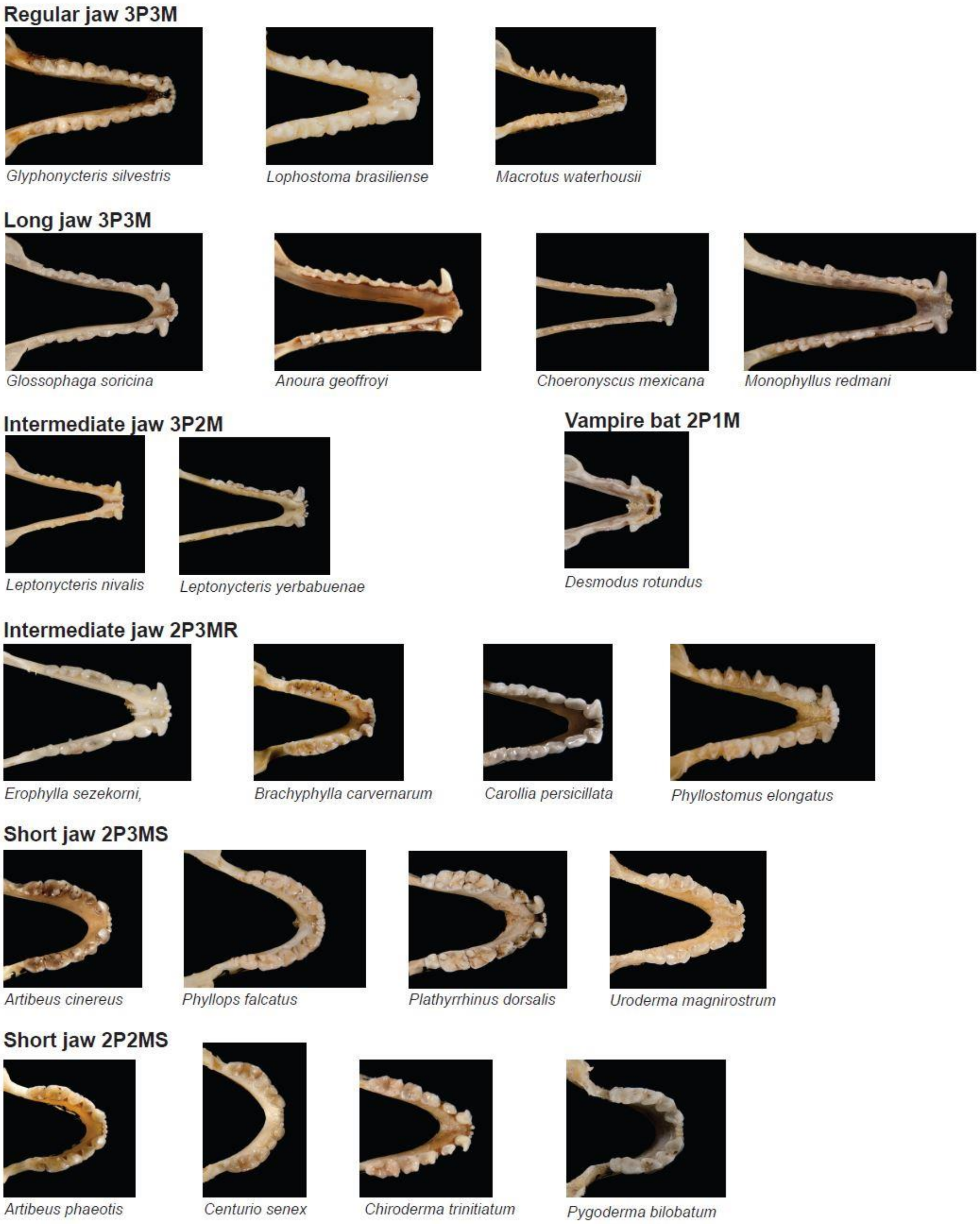

Supplementary Figure 2: Representative bats from the different groups based of tooth number and jaw length in the different morphogroups.
Pictures from ADW (animaldiversity.org, Phil Myer).

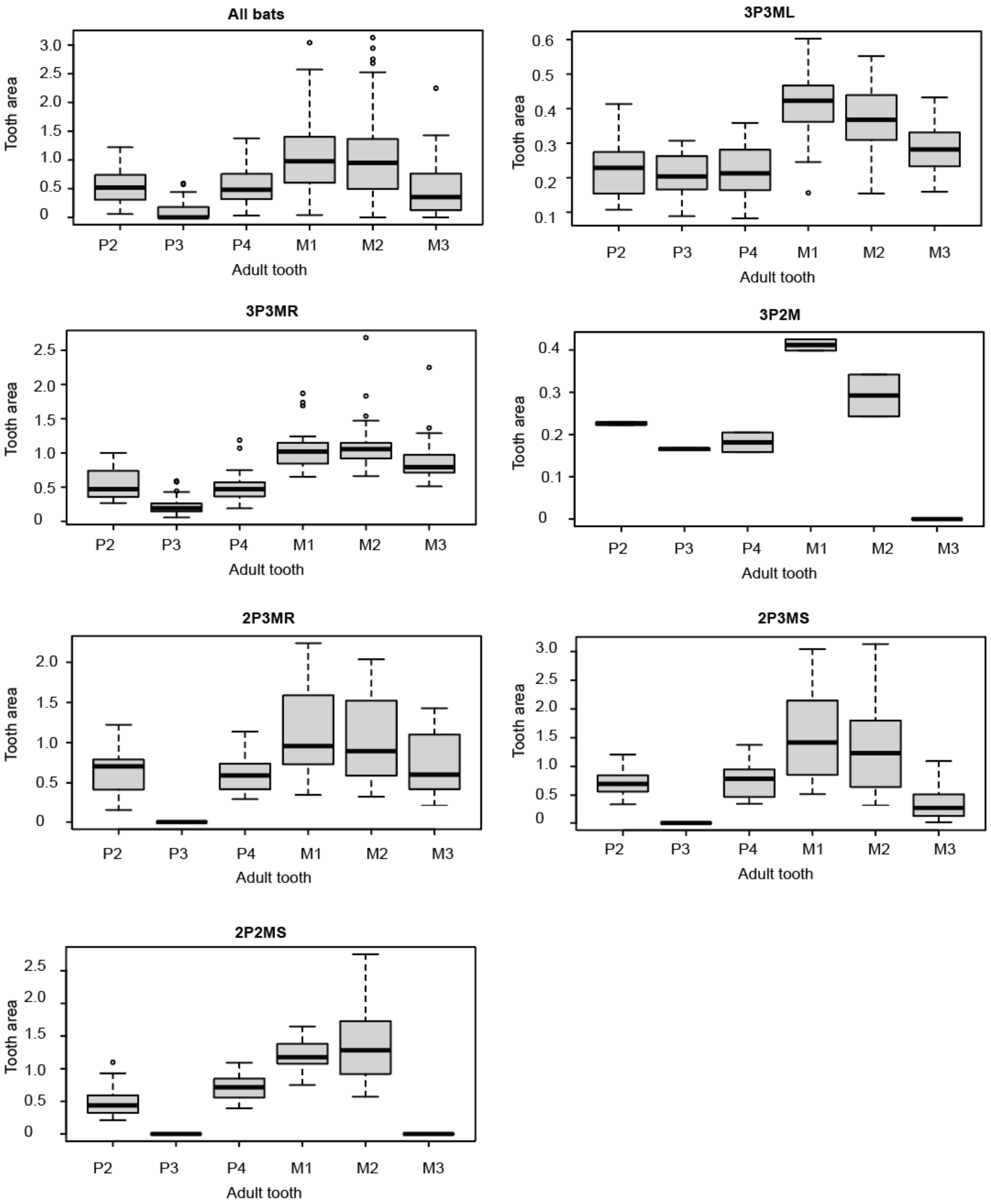

Supplementary Figure 3: Tooth proportion variation in the different groups of bats
Tooth proportions (length x width) variation in the different groups of bats (see Methods). As jaw length diminishes, molar proportions become less equal, in particular for 2P3MS (short jaw). Most of the variation in terms of M1 and M2 proportions in all bats is explained by the 2P2MS (short

jaw) group. Tooth sizes are normalized by adult body mass (Supplementary Data 2). Replicates per species are indicated in the Source Data File 1 (n=2, 3 or 4 biological replicates per species depending on specimen availability in the museum collections, when only one specimen is available (*e.g. Vampyressa thyone*, technical replicate (mean of the left and right side) have been measured).
Boxplot values: Med: median (middle line), First quart: 25th, Third quart: 75th percentile (box) and Min (minimum) and Max (maximum) for the whiskers as well as outliers (single points).

**All bats**	P2	P3	P4	M1	M2	M3
Min	0.059647	0	0.033321	0.039742	0	0
First quart.	0.307427	0	0.319316	0.602685	0.495887	0.124221
Med	0.517796	0	0.481088	0.977045	0.950188	0.353891
Third quart.	0.74164	0.182227	0.759373	1.40165	1.365488	0.76204
Max	1.221781	0.441753	1.375643	2.574	2.524649	1.427744

**3P3ML**	P2	P3	P4	M1	M2	M3
Min	0.107255	0.088729	0.082158	0.245286	0.154388	0.159044
First quart.	0.154098	0.165693	0.163818	0.361687	0.309159	0.23293
Med	0.228468	0.203623	0.212615	0.422795	0.367834	0.281756
Third quart.	0.274629	0.262301	0.280887	0.466795	0.439345	0.331173
Max	0.413225	0.307025	0.358595	0.602685	0.552279	0.432244

**3P3MR**	P2	P3	P4	M1	M2	M3
Min	0.266948	0.058048	0.191429	0.650089	0.660582	0.512434
First quart.	0.358231	0.146021	0.363351	0.844033	0.919915	0.711623
Med	0.471771	0.192104	0.470064	1.019139	1.055401	0.793551
Third quart.	0.739416	0.260809	0.569862	1.148274	1.14676	0.971312
Max	0.99954	0.429455	0.748759	1.242152	1.472036	1.288855

**3P2M**	P2	P3	P4	M1	M2	M3
Min	0.222463	0.163221	0.158257	0.398352	0.24266	0
First quart.	0.222463	0.163221	0.158257	0.398352	0.24266	0
Med	0.226015	0.165707	0.181614	0.411709	0.292301	0
Third quart.	0.229567	0.168193	0.204971	0.425066	0.341941	0
Max	0.229567	0.168193	0.204971	0.425066	0.341941	0

**2P3MR**	P2	P3	P4	M1	M2	M3
Min	0.14979	0	0.297877	0.348944	0.326059	0.214187
First quart.	0.414293	0	0.420761	0.730238	0.588282	0.421181
Med	0.704905	0	0.591317	0.957585	0.894194	0.601776
Third quart.	0.791273	0	0.737339	1.589999	1.522814	1.100803
Max	1.221781	0	1.136969	2.237881	2.036964	1.427744

**2P3MS**	P2	P3	P4	M1	M2	M3

Min	0.341036	0	0.350243	0.517918	0.319502	0.013418
First quart.	0.560873	0	0.468052	0.852508	0.640733	0.121587
Med	0.695479	0	0.785564	1.416291	1.231329	0.265034
Third quart.	0.845608	0	0.948147	2.145266	1.797164	0.514425
Max	1.204052	0	1.375643	3.040636	3.128169	1.09279

**2P2MS**	P2	P3	P4	M1	M2	M3
Min	0.212066	0	0.394046	0.750114	0.571031	0
First quart.	0.323311	0	0.556945	1.07532	0.916973	0
Med	0.437654	0	0.715251	1.175817	1.282368	0
Third quart.	0.592095	0	0.849533	1.381886	1.728577	0
Max	0.929737	0	1.091067	1.646061	2.753693	0

Source data are provided as Source Data File 2.

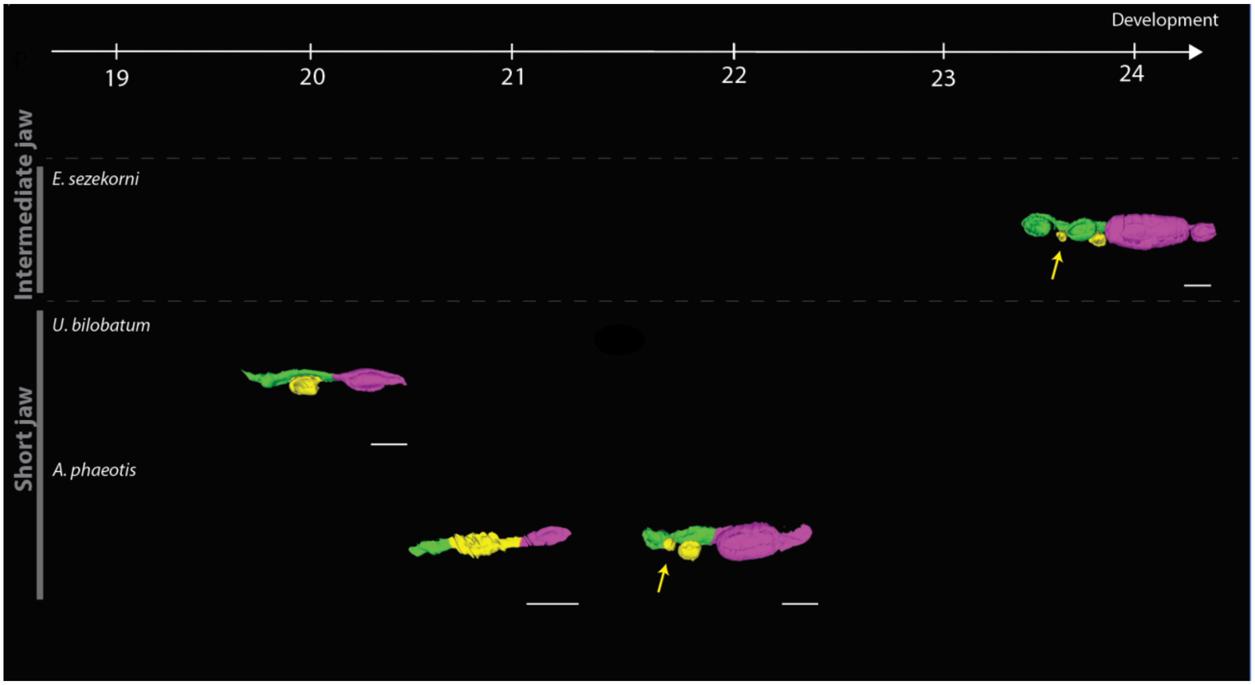

Supplementary Figure 4: µCT scan on isolated stages for more species

Reconstruction of the developing dental lamina in three other species of bats from other morphogroups for which the developmental series is incomplete. Permanent premolars are indicated in green, deciduous premolars in yellow and molars in pink. For *E. sezekorni*, the dP3 is indicated with a yellow arrow and will give rise to a functional dP3. For *U. bilobatum* and *A. phaeotis*, the developing dP3 is indicated with a yellow arrow at its earlier stages but then degenerates before its eruption in the juveniles. Scale bar: 500 µm

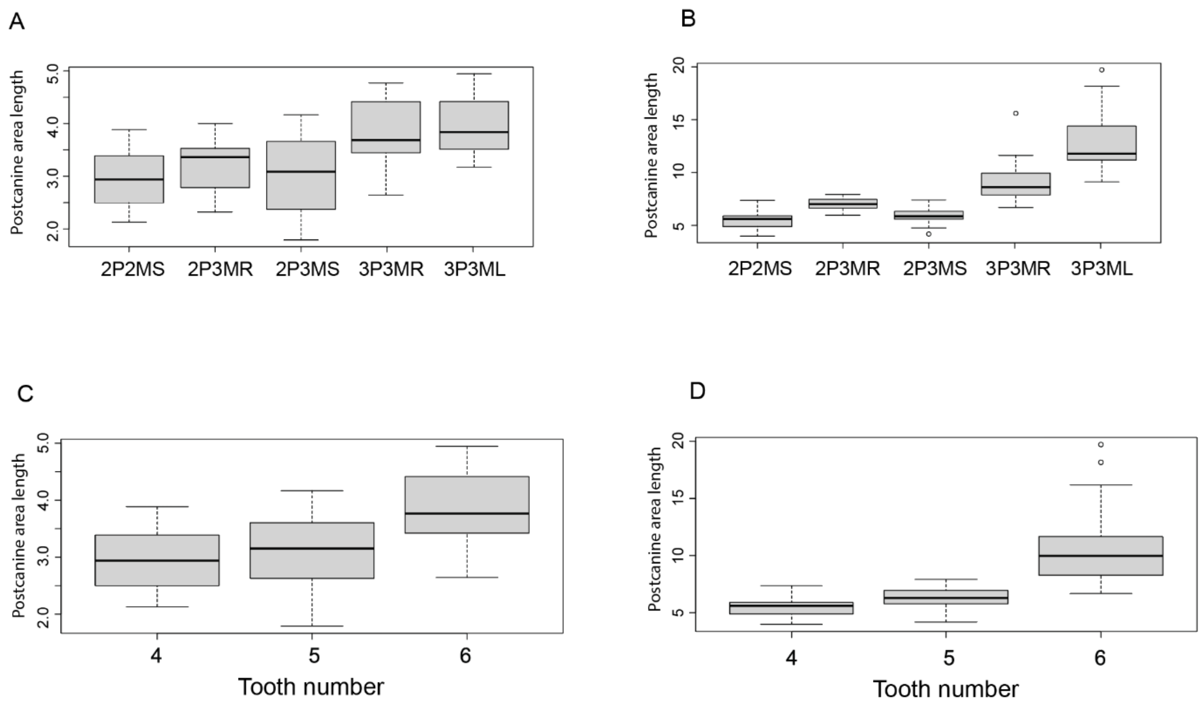

Supplementary Figure 5: Relation between tooth number, dental field and jaw length.
A, B: Dental field variation in the different groups broken down by their dental formula normalized by BM (A) or P4 area (B).
C, D: Dental field variation for different number of teeth normalized by BM (A) or P4 area (B).
Replicates per species are indicated in the Source Data File 2 (n=2, 3 or 4 biological replicates per
E, F: Jaw length variation in the different groups broken down by their dental formula normalized by BM (A) or P4 area (B).
G, H: Jaw length variation for different number of teeth normalized by BM (A) or P4 area (B).
Replicates per species are indicated in the Source Data File 2 (n=2, 3 or 4 biological replicates per species depending on specimen availability in the museum collections, when only one specimen is available (*e.g. Vampyressa thyone*, technical replicate (mean of the left and right side) have been measured).
Boxplot values: Med: median (middle line), First quart: 25th, Third quart: 75th percentile (box) and Min (minimum) and Max (maximum) for the whiskers as well as outliers (single points).

**A**	**2P2MS**	**2P3MS**	**2P3MR**	**3P3MR**	**3P3ML**
Min	2.129418	2.32052	1.790449	3.1733	2.643629
First quart.	2.498388	2.783265	2.372177	3.512884	3.443717
Med	2.939125	3.362353	3.085511	3.838903	3.686285
Third quart.	3.388817	3.529003	3.660785	4.417571	4.414596
Max	3.885555	3.999605	4.166667	4.94514	4.768646

B	2P2MS	2P3MS	2P3MR	3P3MR	3P3ML
Min	3.984687	5.958168	4.749434	9.119117	6.684464
First quart.	4.888605	6.630411	5.589413	11.18507	7.87429
Med	5.601802	7.004767	5.86174	11.77569	8.613386
Third quart.	5.902549	7.467859	6.333642	14.39971	9.93808
Max	7.365895	7.919248	7.397792	18.16057	11.61752

C	4	5	6
Min	2.129418	1.790449	2.643629
First quart.	2.498388	2.627724	3.422058
Med	2.939125	3.152368	3.765494
Third quart.	3.388817	3.604618	4.415487
Max	3.885555	4.166667	4.94514

D	4	5	6
Min	3.984687	4.181269	6.684464
First quart.	4.888605	5.776271	8.280695
Med	5.601802	6.28815	9.970001
Third quart.	5.902549	6.948102	11.66494
Max	7.365895	7.919248	16.17493

Source data are provided as Source Data File 2. Vampire bats (1P2M, 2P2M) and 2P3M bats have been excluded from the analysis.

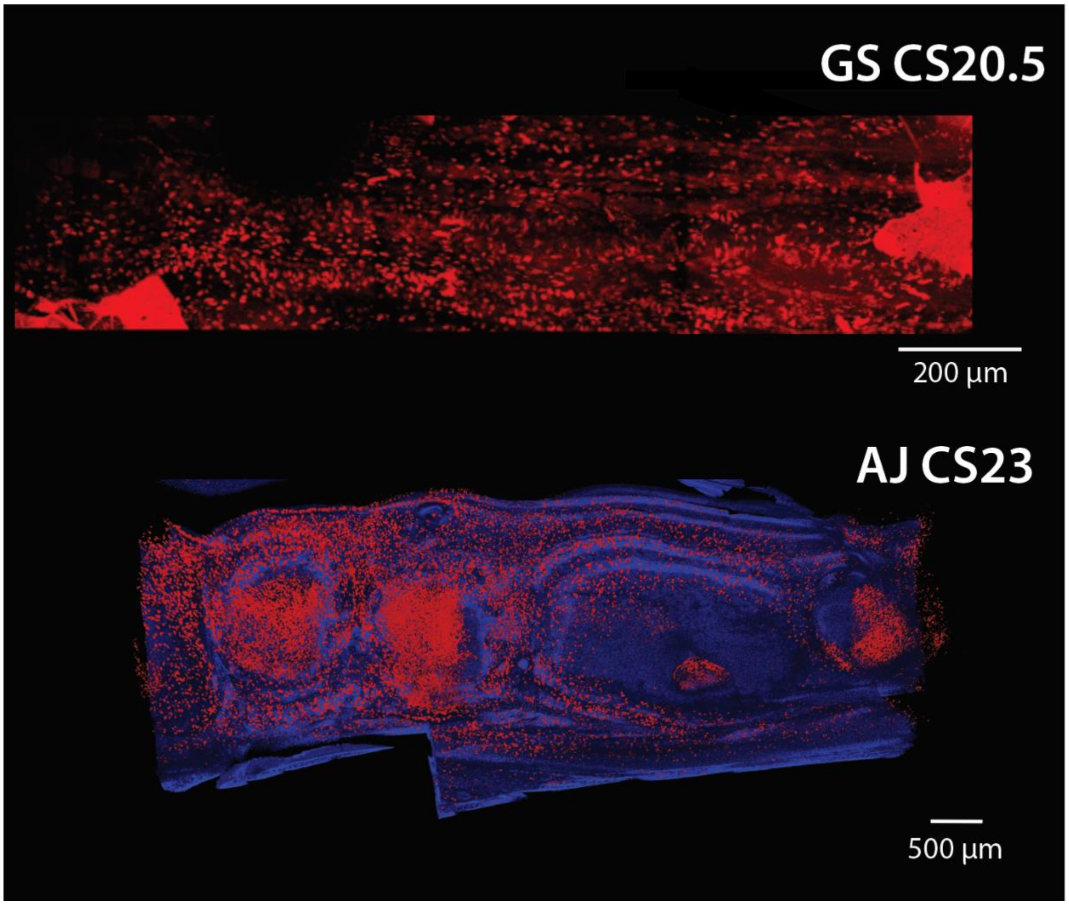

Supplementary Figure 6: EdU from two-photon imaging

Additional species and stages (GS CS20.5: *G. soricina*, carnegie stage 20.5 – scale bar 200 µm; AJ CS23 – scale bar 500 µm: *A. jamaicensis*, carnegie stage 23). This experiment has been performed twice with similar results.

Morphogroups/BM				
**Pairwise comparison**	**diff**	**lwr**	**upr**	**p adj**
**2P3MR-2P2MS**	0.25171249	-0.33546	0.838885	0.757465
**2P3MS-2P2MS**	0.10712009	-0.40903	0.623274	0.978348
**3P3ML-2P2MS**	0.97480036	0.402807	1.546793	6.66E-05
**3P3MR-2P2MS**	0.89166805	0.366691	1.416646	7.11E-05
**2P3MS-2P3MR**	-0.1445924	-0.65052	0.36134	0.932166
**3P3ML-2P3MR**	0.72308787	0.160302	1.285874	0.004847
**3P3MR-2P3MR**	0.63995557	0.125025	1.154886	0.007071
**3P3ML-2P3MS**	0.86768027	0.379448	1.355913	2.91E-05
**3P3MR-2P3MS**	0.78454797	0.352348	1.216748	1.87E-05
**3P3MR-3P3ML**	-0.08313231	-0.58068	0.41442	0.990388
**Morphogroups/P4**				
**Pairwise comparison**	**diff**	**lwr**	**upr**	**p adj**
**2P3MR-2P2MS**	1.508548	-0.01729	3.034386	0.054227
**2P3MS-2P2MS**	0.45074	-0.89055	1.792027	0.883723
**3P3ML-2P2MS**	7.506366	6.019974	8.992757	0
**3P3MR-2P2MS**	3.563341	2.199125	4.927558	0
**2P3MS-2P3MR**	-1.057808	-2.37253	0.256916	0.175825
**3P3ML-2P3MR**	5.997818	4.535351	7.460285	0
**3P3MR-2P3MR**	2.054793	0.716684	3.392902	0.000415
**3P3ML-2P3MS**	7.055626	5.786896	8.324356	0.0000000
**3P3MR-2P3MS**	3.112601	1.989479	4.235723	0
**3P3MR-3P3ML**	-3.943025	-5.23597	-2.65008	0
**Tooth number/BM**				
**Pairwise comparison**	**diff**	**lwr**	**upr**	**p adj**
**4-5**	0.1572848	-0.25619	0.570758	0.639081
**4-6**	0.9245746	0.510042	1.339107	1.8E-06
**5-6**	0.7672898	0.475671	1.058909	0
**Tooth number/P4**				
**Pairwise comparison**	**diff**	**lwr**	**upr**	**p adj**
**4-5**	0.8177346	-0.58143	2.216902	0.350384
**4-6**	5.124122	3.721371	6.526873	0
**5-6**	4.3063873	3.319566	5.293208	0

Supplementary Table 1: Comparison between the size of the dental field and dental formula. Tukey HSD test (single-step multiple comparisons of means, one-sided) of morphogroups dental field lengths normalized by body mass or P4 area. This test compares the difference between each pair of means with appropriate adjustment for the multiple testing. 2P2MS morphogroup significantly different from 3P3ML and 3P3MR. 2P3MR morphogroup is significantly different from 3P3MR and 3P3ML. 2P3MS morphogroup is significantly different from 3P3ML and 3P3MR. Tukey multiple comparisons of means of tooth number dental field lengths normalized by body mass or P4 area. Dental field length of jaws with 4 or 5 postcanine teeth are significantly smaller than jaws with 6 teeth.

Source Data are provided as Source Data File 2. Vampire bats (1P2M, 2P2M) and 2P3M bats have been excluded from the analysis.

Supplementary Note 1: Mathematical Modelling Supplementary Information for *Bat teeth illuminate the diversification of mammalian tooth classes*

1 Overview of Supplement

Here we propose a simple reaction-diffusion model of the formation and organisation of bat teeth primordia as the jaw grows. Between different species the size of the teeth and jaw, as well as the number and sequence of appearance of each bud, vary substantially. From its original appearance, these buds become the signalling centre of each tooth, and do not change as the jaw extends in each direction.

We will first consider sequences (in time) of insertions of each tooth bud, given by where it first appears in relation to the other teeth primordia. SI Figure 1 shows the experimentally-obtained sequences of insertions which come in four prototypical types across the observed species, though morphology, jaw length, and other details may change between each species even if the sequence of insertions is the same. The modelling presented here will be qualitative – as we do not know the details of the underlying molecular kinetics, we instead aim to provide examples where a simple reaction-diffusion theory can capture different sequences of tooth bud insertions over time, changing only aspects of the domain growth.

In Section 2 we present a simple reaction-diffusion model on a static domain to use as a basic framework for pattern formation, and discuss aspects of internal localisation of spots, representing the tooth buds. In Section 3 we extend this framework to include non-uniform domain growth, and propose a simple way of modelling such growth. We use this model in Section 4 to show how simple differential growth can capture a large variety of the insertion sequences observed, for equally sized teeth primordia. We then show in Subsection 4.3 how spatial heterogeneity (modelling other aspects of cell signalling) can in principle modulate size and spacing of teeth primordia, though at the cost of increasing model complexity (see [11] for discussions on the generality and limitations of such approaches). Finally in Section 5 some closing remarks about extending the basic ideas, particularly with an eye towards challenges in the theory, are made.

2 Model in Static Domains (No Growth)

Here we present the basic model, using the classical Gierer-Meinhardt kinetics [2]. In the absence of detailed knowledge about the underlying signalling network, we consider a presumptive activator u and inhibitor v that obey the following system of reaction-diffusion equations,

$$\frac{\partial u}{\partial t} = \underbrace{\nabla^2 u}_{\text{Diffusion}} + \underbrace{\gamma + \frac{u^2}{v}}_{\text{Production}} - \underbrace{au}_{\text{Degradation}}, \tag{1}$$

$$\frac{\partial v}{\partial t} = \underbrace{d\nabla^2 v}_{\text{Diffusion}} + \underbrace{u^2}_{\text{Production}} - \underbrace{bv}_{\text{Degradation}}, \tag{2}$$

where γ is a background or basal production of the activator, a and b are the degradation rates of the activator and inhibitor respectively, d is proportional to the ratio of diffusivities, and ∇^2 represents the Laplacian (diffusion) in a domain $\boldsymbol{x} \in \Omega \subseteq \mathbb{R}^n$, where we will consider two and three dimensional domains, so $n = 2, 3$. We remark that these equations are non-dimensonalized, so that these four parameters are really groupings of dimensional parameters specific to the biochemistry involved. The non-dimensionalized form is taken such that the lengthscale

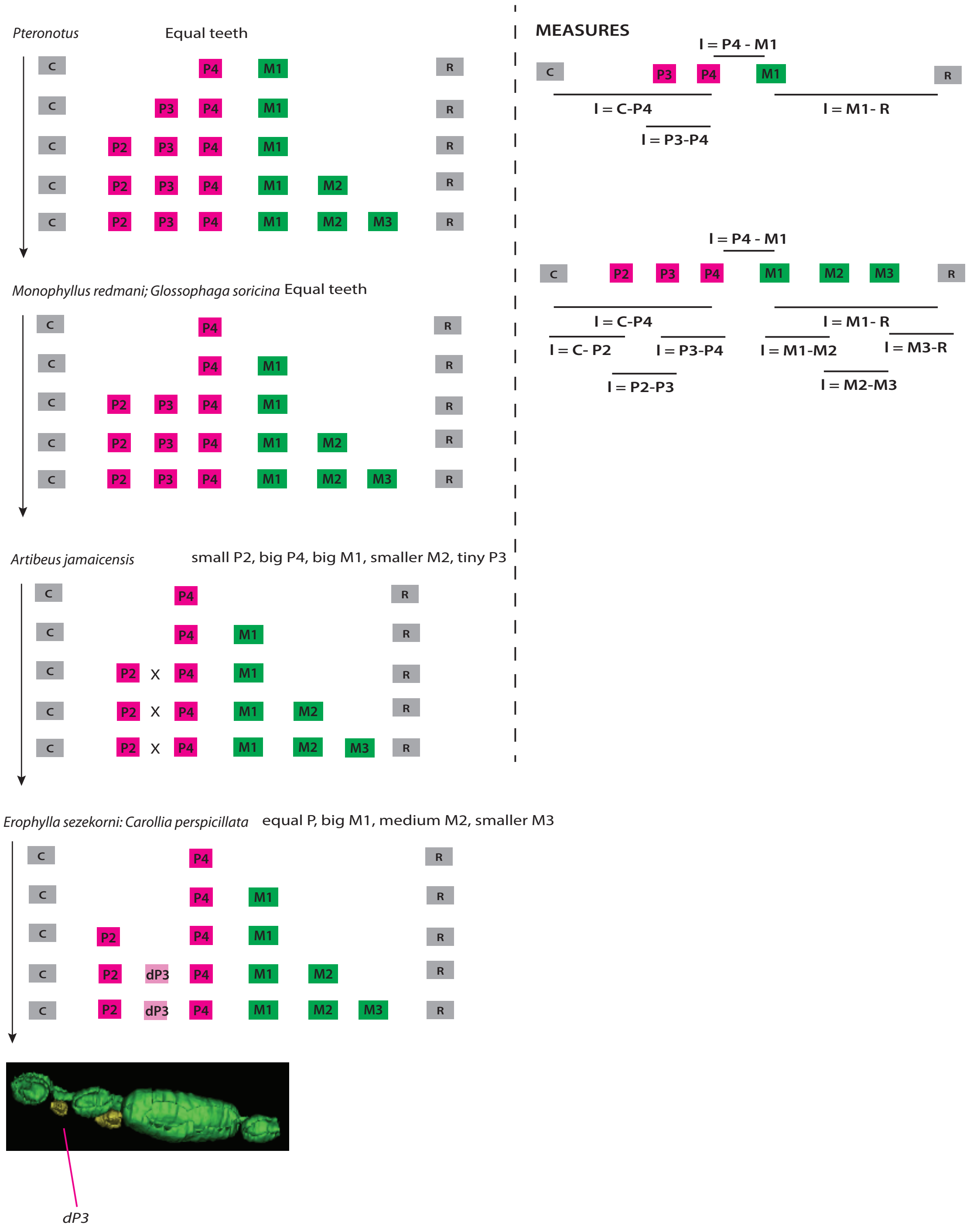

SI Figure 1: Sequence of primordia insertions as the jaw evolves across four different bat species, with time going downwards in each of the four panels.

of the domain L is in units comparable to the activator's diffusion, which is taken to unity. Again, as we lack precise data on the presumptive morphogens, these may represent individual proteins, large signalling networks, or even cells in different stages of differentiation. Hence the model is not meant to capture quantitative details, but to test qualitative hypotheses of what underlies differences in morphology between species.

This system has a single spatially homogeneous steady state given by

$$u^* = \frac{b+\gamma}{a}, \quad v^* = \frac{(u^*)^2}{b} = \frac{(b+\gamma)^2}{a^2 b}.$$

We employed the usual Turing-type analysis to find parameters for which this steady state is stable in the absence of diffusion, but unstable to spatial perturbations when diffusion is considered, as these will typically lead to patterned solutions [8, 4]. In two and three spatial dimensions, within the parameter regimes identified above, the Gierer-Meinhardt system will generically form periodically arranged spots. The precise amplitude, wavelength, and arrangement of these spots depends on both the kinetic and diffusion parameters, as well as the geometry and boundary conditions. We primarily used mixed boundary conditions of the following form to prevent spots from accumulating near the boundaries:

$$u = 0, \quad \boldsymbol{n} \cdot \nabla v = 0, \quad \text{for all } \boldsymbol{x} \in \partial\Omega, \tag{3}$$

where $\boldsymbol{n}$ is the unit normal out of the domain, and $\partial\Omega$ represents the boundary. See [7] for a biological and mathematical discussion of these boundary conditions. The first of these conditions says that the activator concentration is fixed to zero at the edge of the region of interest (the jaw), and the inhibitor cannot diffuse outside of this region (i.e. a no-flux condition). For comparison in SI Figure 3(a), we also used the Neumann conditions,

$$\boldsymbol{n} \cdot u = 0, \quad \boldsymbol{n} \cdot \nabla v = 0, \quad \text{for all } \boldsymbol{x} \in \partial\Omega. \tag{4}$$

2.1 Numerical Details

This model was simulated using commercial finite element software (Comsol Multiphysics V5.6) on rectangular and rectangular cuboid domains. Convergence checks in space and time were carried out, and specific simulations were also carried out in Matlab using the 5 (in 2D) and 7 (in 3D) point finite difference stencil (to ensure the finite element solutions were converging to the same solutions found via a different approach). In 2D, $50,786$ triangular finite elements were used, and in 3D $23,887$ tetrahedral elements were used, with solutions compared to those on finer meshes in both cases for particular simulations to ensure convergence. Initial data were taken as $u(\boldsymbol{x}, 0) = u^*\xi(\boldsymbol{x})$ and $u(\boldsymbol{x}, 0) = u^*\eta(\boldsymbol{x})$, where ξ and η are normally-distributed random variables (at each spatial grid point) with mean 1 and standard deviation 10^{-2}. Larger and smaller random perturbations had little effect on the pattern sequence, as long as the timescales were sufficiently large. Even exactly homogeneous initial data form identical spatial patterns, as discussed in [7], due to the boundary conditions acting as a spatial heterogeneity. We therefore anticipate these results to be essentially insensitive of the initial conditions used.

2.2 Simulation Results

In SI Figure 2 we plot two examples of static spot formation in two and three dimensional domains. The domain sizes here are fairly arbitrary, and lengthening or shortening them in the lateral direction would lead to more or fewer spots respectively. We also demonstrate the use of thresholding to indicate where plausible cell fate specification boundaries may be. This is particularly useful in the 3-D case for visualization purposes.

In SI Figure 3, we explore the use of Neumann (no-flux) boundary conditions (4) in panel (a), thin domains in (b), and thicker domains in (c). As shown in [7], no-flux boundaries can lead to spot formation along boundaries, where 'half-spots' may form, which is not something observed during bat teeth development but could be explained by other developmental and/or physico-chemical constraints present during the development of bat jaws. Such half-spot patterns motivate the use of the mixed boundary conditions (3). Thin domains using the mixed boundary conditions (3), shown in (b), exhibit elliptical spots rather than circular ones, though appear to be otherwise unchanged from simulations with larger lateral extents. If the lateral extent of the domain becomes too large, however, more than one row of spots may form, as in panel (c). This condition, which is not seen in mammals, is supposed to be due to a restriction of the mammalian dentition to one single row due to a pre-pattern, independently from reaction-diffusion mechanisms [12].

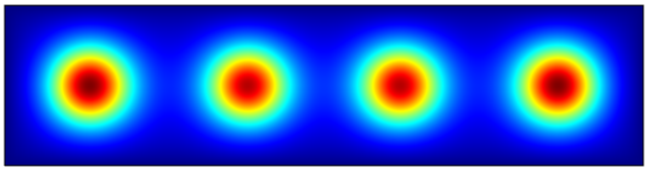

(a)

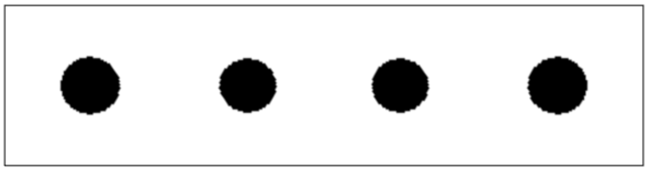

(b)

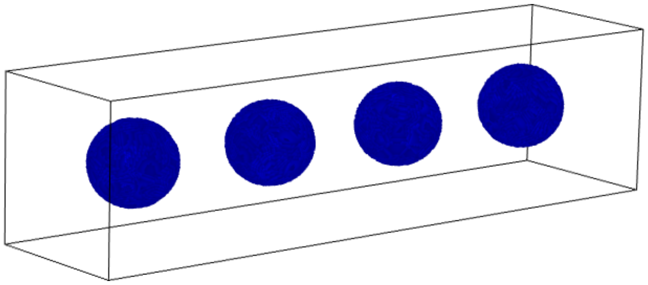

(c)

SI Figure 2: 2-D and 3-D simulations of (1)-(2) with $a = 0.5$, $\gamma = 1$, $b = 5.5$. The 2D plots are in a domain of size 15×60, and the 3D plot is in a domain of size $15 \times 15 \times 60$. (b) and (c) were 'thresholded' so that only the regions where $u > 25$ are shown, indicating a plausible cell fate specification boundary, though the actual concentration diffuses away from these peaks as shown in (a).

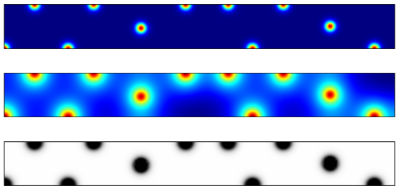

(a) Neumann BCs, $L_y = 2$

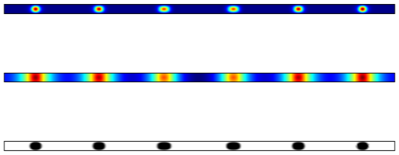

(b) Mixed BCs, $L_y = 0.4$

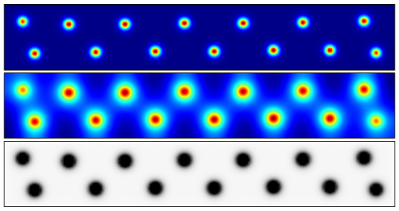

(c) Mixed BCs, $L_y = 3$

SI Figure 3: Simulations of the reaction-diffusion system with different geometries and boundary conditions. We show concentrations of the activator u in the top row, inhibitor v in the middle row, and a thresholded version of the activator in the bottom row where black is $u > u_{\text{Threshold}}$. The parameters used are $L_x = 18$, $L_y = 2$, $a = 0.5$, $b = 1$, $\gamma = 0.1$, $R = 0.3$, and $d = 0.005$. The initial length of the domain is $L_x = 4.5$, and the final length is $5L_x = 22.5$.

3 Non-Uniform Growth Model

Following [1], we formulate a model on a domain with non-uniform growth. We implement this by considering only growth in the x direction, which we will rescale to an initial Lagrangian unit square (the rescaling in the y direction is just a standard scaling, whereas in the x direction the change of coordinates from a growing frame will introduce additional terms). As in [1], we consider a domain with two apically growing segments of possibly different growth rates, $r_1(t)$ and $r_2(t)$ (the first will represent the premolar region, and the second the molar region) according to the observations and mesearments made on μCT scans. We write ξ as the rescaled x coordinate and y rescaled by length L_y (noting that we think of $(\xi, y) \in [0,1]^2$). In the Eulerian frame then the total domain length is $L_x r(t)$ where L_x is the initial length, and $r(t) = 1 + r_1(t)r_2(t)$.

We then have the evolution equations,

$$\frac{\partial u}{\partial t} = d\left(\frac{1}{(L_x r(t))^2}\frac{\partial^2 u}{\partial \xi^2} + \frac{1}{L_y^2}\frac{\partial^2 u}{\partial y^2}\right) + \left(\frac{\xi \dot{r}(t) - \dot{r_1}(t)}{r(t)}\right)\frac{\partial u}{\partial \xi} + \gamma + \frac{u^2}{v} - au, \tag{5}$$

$$\frac{\partial v}{\partial t} = \frac{1}{(L_x r(t))^2}\frac{\partial^2 v}{\partial \xi^2} + \frac{1}{L_y^2}\frac{\partial^2 v}{\partial y^2} + \left(\frac{\xi \dot{r}(t) - \dot{r_1}(t)}{r(t)}\right)\frac{\partial v}{\partial \xi} + u^2 - bv, \tag{6}$$

where we have the additional terms due to growth are given by the advection (first order space derivatives). For apical growth these essentially arise due to a change of coordinate system.

In principle this allows us to describe the non-uniform growth by specifying the two growth functions on each endpoint. More general nonuniform growth functions are difficult to determine, even if mitosis frequency can be measured across the jaw, and in general comparing patterns which form on timescales comparable with growth is quite difficult - much of their characteristics will depend on the nonlinearities chosen, which are impossible to know without a detailed understanding of the biochemical signalling. So we will build up sensible choices of growth functions to qualitatively match domain expansion, under this simple apical growth model.

We use the following smooth function with two parameters:

$$B(t; g, t_j) = \frac{1 + \tanh(g(t - t_j))}{2}, \tag{7}$$

where g is a smoothing coefficient, and t_j is a switching time. This function smoothly goes from 0 for small values of t, to 1 for larger values. Specifically, it is monotonically increasing and for $|g(t - t_j)| > 2.3$, B is within 0.1% of 0 (for $t < t_j$) or 1 (for $t > t_j$). We can then vary g to have the growth occur over longer periods of time, and take linear combinations of these B functions to represent more complex sequences of growth.

4 Numerical Results Matching Sequence of Insertions

We now use the framework described to reconstruct the insertion sequences shown in SI Figure 1.

4.1 Equally-Sized Teeth

We use simple combinations of the growth functions above to match the first three sequences of insertions in SI Figure 1. We aim only to match the sequence of the insertions, noting that quantitative aspects (such as matching a particular species' time in each stage) can be readily tuned.

We start by giving growth functions for *Pteronatus quadridens* in SI Figure 4(a), and show the sequence of insertions in SI Figure 5. As noted, the precise timings can be tuned by properly tuning the growth functions. We show sequences of insertions for *Monophyllus redmani* and *Glossophaga soricina* in SI Figure 6, using the growth functions given in SI Figure 4(b). Note that in SI Figure 6, we have included a snapshot of P3 forming near $t = 0.3T_f$, . The time frame where P3 exists but P2 does not can be made arbitrarily small with more rapid growth of $r_1(t)$ In SI Figure 7, we give the insertion sequence for *Artibeus jamaicensis*, in the absence of P3 and assuming that the primordia are equally sized and spaced.

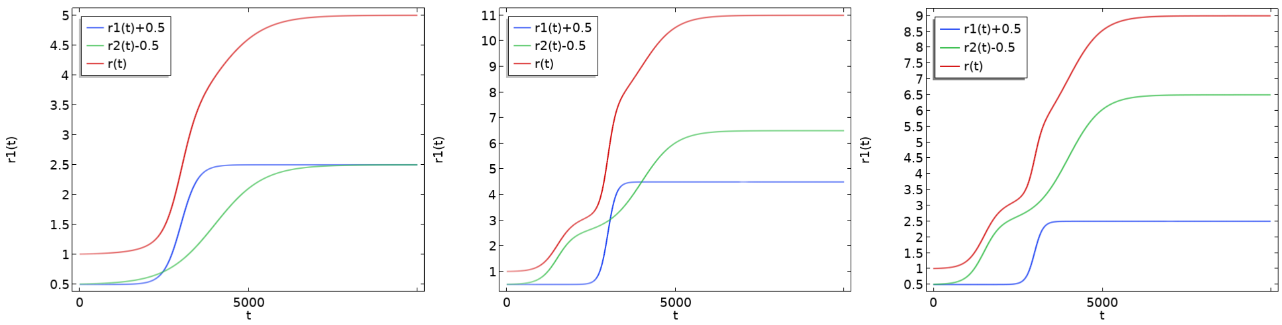

SI Figure 4: Growth functions in (a) for the domain given by $r_1(t) = 2B(t/T_f; 20, 0.3T_f)$, $r_2(t) = 1 + 2B(t/T_f; 7, 0.4T_f)$ in (b) for the domain given by $r_1(t) = 4B(t/T_f; 40, 0.3T_f)$, $1 + 4B(t/T_f; 10, 0.4T_f) + 2B(t/T, 20, 0.15T_f)$ and in (c) for the domain given by $r_1(t) = 2B(t/T_f; 40, 0.3T_f)$, where $T_f = 10^4$ is a final timescale, and $r(t) = r_1(t) + r_2(t)$. Note the shift by 0.5 in the plots is to center the domain at $r_1(0)$ and $r_2(0)$ so that $r_1(t)$ and $r_2(t)$ can be compared as t increases.

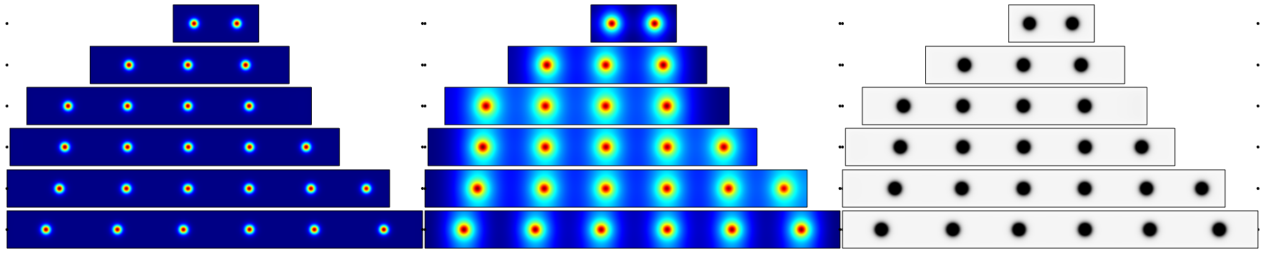

(a) u (b) v (c) $u > u_{\text{Threshold}} = 2$

SI Figure 5: Simulations of the reaction-diffusion system (5)-(6) with the evolution of the domain given in SI Figure 4(a). We show concentrations of the activator in (a), inhibitor in (b), and a thresholded version of the activator in (c). The top row is shown at time $T = 0.1T_f$, where $T_f = 10^4$ is a final timescale, and each subsequent row is shown at $T = 0.3T_f$, $T = 0.35T_f$, $T = 0.4T_f$, $T = 0.5T_f$, and $T = T_f$. The parameters used are $L_x = 4.5$, $L_y = 2$, $a = 0.5$, $b = 1$, $\gamma = 0.1$, $R = 0.3$, and $d = 0.005$. The initial length of the domain is $L_x = 4.5$, and the final length is $5L_x = 22.5$.

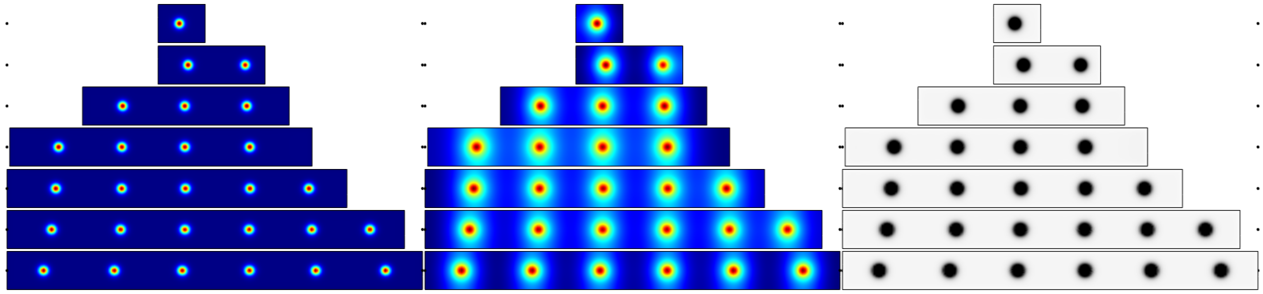

(a) u (b) v (c) $u > u_{\text{Threshold}} = 2$

SI Figure 6: Simulations of the reaction-diffusion system (5)-(6) with the evolution of the domain given in SI Figure 4(b). We show concentrations of the activator in (a), inhibitor in (b), and a thresholded version of the activator in (c). The top row is shown at time $T = 0.1T_f$, where $T_f = 10^4$ is a final timescale, and each subsequent row is shown at $T = 0.2T_f$, $T = 0.35T_f$, $T = 0.4T_f$, $T = 0.5T_f$, and $T = T_f$. The parameters used are $L_x = 2$, $L_y = 2$, $a = 0.5$, $b = 1$, $\gamma = 0.1$, $R = 0.3$, and $d = 0.005$. The initial length of the domain is $L_x = 2$, and the final length is $10L_x = 20$.

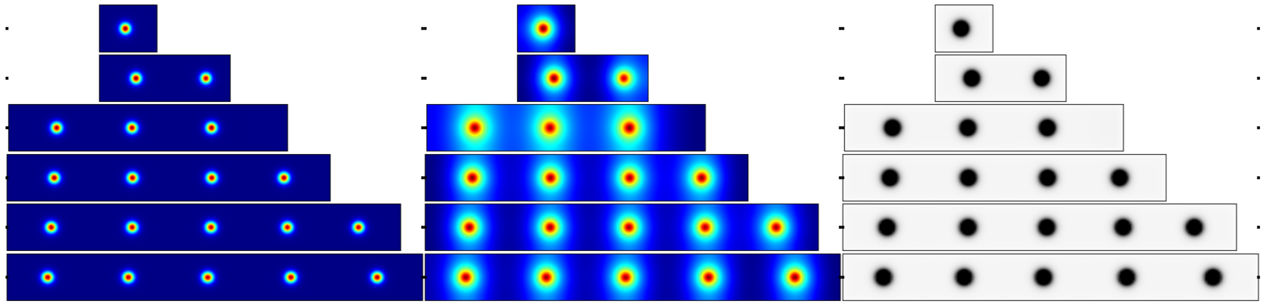

(a) u (b) v (c) $u > u_{\text{Threshold}} = 2$

SI Figure 7: Simulations of the reaction-diffusion system (5)-(6) with the evolution of the domain given in SI Figure 4(c). We show concentrations of the activator in (a), inhibitor in (b), and a thresholded version of the activator in (c). The top row is shown at time $T = 0.1T_f$, where $T_f = 10^4$ is a final timescale, and each subsequent row is shown at $T = 0.2T_f$, $T = 0.35T_f$, $T = 0.4T_f$, $T = 0.5T_f$, and $T = T_f$. The parameters used are $L_x = 2$, $L_y = 2$, $a = 0.5$, $b = 1$, $\gamma = 0.1$, $R = 0.3$, and $d = 0.005$. The initial length of the domain is $L_x = 2$, and the final length is $9L_x = 18$.

4.2 Non-Apical Domain Growth Leads to Internal Insertions

In the fourth set of observed cases (*Erophylla sezekomi* and *Carollia perspicillata*), as well as the creation of new bud elements at the ends of the growing jaw, we also have the insertion of dP3 between two pre-existing buds. If we assume the domain growth is non-uniform, but also not purely apical, we can obtain a sequence of insertions matching this case. Again following [1], we consider a Lagrangian domain which is a unit square, and assume uniform growth at a rate $r_1(t)$ for $x \in [0, \theta)$, and uniform growth at a rate $r_2(t)$ for $x \in [\theta, 1]$. The total domain length in the x direction at time t is then given by $L_x r(t) = L_x(r_1(t)\theta + r_2(t)(1-\theta))$.

We can then write the equations of motion in the Lagrangian frame as,

$$\frac{\partial u}{\partial t} = d\left(\frac{1}{(L_x r(t))^2}\frac{\partial^2 u}{\partial \xi^2} + \frac{1}{L_y^2}\frac{\partial^2 u}{\partial y^2}\right) + \mathcal{B}(x)\frac{\partial u}{\partial \xi} + \gamma + \frac{u^2}{v} - au - 2S(x)u, \tag{8}$$

$$\frac{\partial v}{\partial t} = \frac{1}{(L_x r(t))^2}\frac{\partial^2 v}{\partial \xi^2} + \frac{1}{L_y^2}\frac{\partial^2 v}{\partial y^2} + \mathcal{B}(x)\frac{\partial v}{\partial \xi} + u^2 - bv - 2S(x)u, \tag{9}$$

where the functions S and $\mathcal{B}$ are defined by,

$$S(x) = \begin{cases} \dfrac{\dot r_1(t)}{r_1(t)}, & \xi < \theta \\ \dfrac{\dot r_2(t)}{r_2(t)}, & \xi \geq \theta \end{cases}, \quad \mathcal{B}(x) = \begin{cases} \xi\left(\dfrac{\dot r(t)}{r(t)} - \dfrac{\dot r_1(t)}{r_1(t)}\right), & \xi < \theta \\ \xi\left(\dfrac{\dot r(t)}{r(t)} - \dfrac{\dot r_2(t)}{r_2(t)}\right) - \theta\left(\dfrac{\dot r_1(t)}{r_1(t)} - \dfrac{\dot r_2(t)}{r_2(t)}\right), & \xi \geq \theta \end{cases}. \tag{10}$$

We note that the S term represents a dilution of morphogen due to the growth, which is neglected in the apical case as there the growth is so localized that no dilution occurs. The advection term, given by $\mathcal{B}$, is also more complicated in this case.

We plot the morphogen concentrations from our simulations in SI Figure 8. We remark that, besides the form of the growth and the final domain size, the parameters are identical to the other cases considered. Here the final domain is larger (so the buds appear smaller), as the dilution of concentration leads to overall fewer insertions on the timescale of growth. We observe the same sequence of insertions as given in the fourth case of SI Figure 1. Of course this sequence of insertions could be obtained in other ways by tuning parameters, or considering more complicated combinations of apical and locally-uniform regions of growth, but our aim here is to demonstrate that differential growth alone is sufficient to recapitulate these sequences.

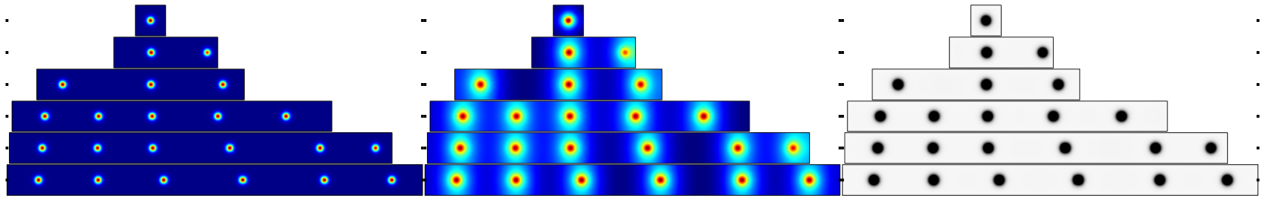

(a) u (b) v (c) $u > u_{\text{Threshold}} = 2$

SI Figure 8: Simulations of the reaction-diffusion system (8)-(10) with the evolution of the domain given by $r_1(t) = 1 + 8.5B(t/T_f; 20, 0.3T_f)$ and $r_2(t) = 1 + 12B(t/T_f, 40, 0.38T_f) + 5B(t/T_f, 40, 0.25T_f)$. We show concentrations of the activator in (a), inhibitor in (b), and a thresholded version of the activator in (c). The top row is shown at time $T = 0.1T_f$, where $T_f = 10^4$ is a final timescale, and each subsequent row is shown at $T = 0.26T_f$, $T = 0.33T_f$, $T = 0.38T_f$, $T = 0.4T_f$, and $T = T_f$. The parameters used are $L_x = 2$, $L_y = 2$, $a = 0.5$, $b = 1$, $\gamma = 0.1$, $R = 0.3$, and $d = 0.005$. The initial length of the domain is $L_x = 2$, and the final length is $13.75L_x = 18$.

4.3 Heterogeneity Allows for Variation in Teeth Sizes

As shown above, all of the localized solutions (spots) observed in SI Figures 5-7 are approximately equally sized and spaced as the domain evolves. That is, they are all roughly circular and equally spaced, though the precise spacing can vary slightly as the domain expands (this can be understood as the domain is between two integer numbers of spots being permissible). Following studies of size and wavelength modulation in [9, 10] numerically and [6, 3] analytically, the authors in [11] demonstrated how simple spatial heterogeneities could be used to design relatively complex patterns. Here, we will introduce a simple modification of (5)-(6) in order to obtain sequences of primordia with different sizes.

We introduce the function,

$$A(\xi) = c_1 - c_2 \cos(\pi(\xi - c_3)), \tag{11}$$

which we use to modulate the linear degradation of the activator, u. This leads us to the equations,

$$\frac{\partial u}{\partial t} = d\left(\frac{1}{(L_x r(t))^2}\frac{\partial^2 u}{\partial \xi^2} + \frac{1}{L_y^2}\frac{\partial^2 u}{\partial y^2}\right) + \left(\frac{\xi \dot{r}(t) - \dot{r_1}(t)}{r(t)}\right)\frac{\partial u}{\partial \xi} + \gamma + \frac{u^2}{v} - aA(\xi)u, \tag{12}$$

$$\frac{\partial v}{\partial t} = \frac{1}{(L_x r(t))^2}\frac{\partial^2 v}{\partial \xi^2} + \frac{1}{L_y^2}\frac{\partial^2 v}{\partial y^2} + \left(\frac{\xi \dot{r}(t) - \dot{r_1}(t)}{r(t)}\right)\frac{\partial v}{\partial \xi} + u^2 - bv, \tag{13}$$

which are identical to (5)-(6) except for the term $aA(\xi)u$.

In SI Figure 9, we show a revised version of SI Figure 7 incorporating spatial heterogeneity in the activator kinetics as in (12)-(13), but using the same growth conditions as in SI Figure 5(c). We set $c_1 = 1.3$, $c_2 = 0.9$, and $c_3 = 0.4$. By design, P4 and M1 are larger, whereas P2 and M2 (again assuming P3 is absent) are smaller, and M3 is smaller still. We note that qualitatively similar patterns can be obtained with different values (or tweaking the 'base' kinetic parameters), particularly to exaggerate these size differences. However we note two barriers to this in general. Firstly, heterogeneity will typically modulate the wavelength between spots, and especially on growing domains this can lead to exaggerated spot movement (note that M1, M2, and M3 all emerge from the right boundary as very small spots before moving closer and becoming larger). Secondly, nonlinear effects can lead to complex spatiotemporal oscillations, and other bifurcations breaking the simple symmetries observed in the spatially homogeneous examples; see [5] for further discussion of this point.

5 Discussion

We have demonstrated how a very simple reaction-diffusion mechanism can produce most of the insertion sequences observed under a simple model of an apically growing domain. We have also shown that tuning kinetic parameters via spatial heterogeneity can match aspects of tooth modulation, at the cost of adding additional parameters and functions to the model, and influencing the wavelength (spacing) of teeth primordia. Of course,

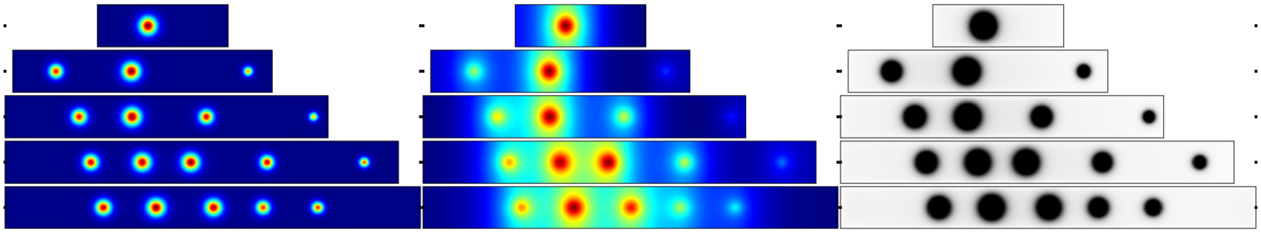

SI Figure 9: Simulations of the reaction-diffusion system (12)-(13) with the evolution of the domain given in SI Figure 4(c). We show concentrations of the activator in (a), inhibitor in (b), and a thresholded version of the activator in (c). The top row is shown at time $T = 0.2T_f$, where $T_f = 10^4$ is a final timescale, and each subsequent row is shown at $T = 0.33T_f$, $T = 0.4T_f$, $T = 0.5T_f$, and $T = T_f$. The parameters used are $L_x = 1.8$, $L_y = 2$, $a = 0.5$, $b = 1$, $\gamma = 0.1$, $R = 0.3$, and $d = 0.005$. The initial length of the domain is $L_x = 1.8$, and the final length is $9L_x = 18$.

such modulation may actually occur via cell-fate specification downstream of a reaction-diffusion process which only fixes the signalling centres of each bud. Finally we also note that geometry can modulate these spot patterns, so that for thinner domains, elliptical patterns are obtained (see SI Figure 5(b)).

References

[1] EJ Crampin, WW Hackborn, and PK Maini. Pattern formation in reaction-diffusion models with nonuniform domain growth. *Bulletin of mathematical biology*, 64(4):747–769, 2002.

[2] A. Gierer and H. Meinhardt. A theory of biological pattern formation. *Kybernetik*, 12(1):30–39, 1972.

[3] Michal Kozák, Eamonn A Gaffney, and Václav Klika. Pattern formation in reaction-diffusion systems with piecewise kinetic modulation: An example study of heterogeneous kinetics. *Physical Review E*, 100(4):042220, 2019.

[4] A. L. Krause, E. A. Gaffney, P. K. Maini, and V Klika. Modern perspectives on near-equilibrium analysis of Turing systems. *Philosophical Transactions of the Royal Society A: Mathematical, Physical and Engineering Sciences*, 379(2213), 2021.

[5] A. L. Krause, V. Klika, T. E. Woolley, and E. A. Gaffney. Heterogeneity induces spatiotemporal oscillations in reaction-diffusion systems. *Physical Review E*, 97(5):052206, 2018.

[6] A. L. Krause, V. Klika, T. E. Woolley, and E. A. Gaffney. From one pattern into another: Analysis of Turing patterns in heterogeneous domains via WKBJ. *Journal of the Royal Society Interface*, 17:20190621, 2020.

[7] Andrew L Krause, Václav Klika, Philip K Maini, Denis Headon, and Eamonn A Gaffney. Isolating patterns in open reaction–diffusion systems. *Bulletin of Mathematical Biology*, 83(7):1–35, 2021.

[8] J. D. Murray. *Mathematical Biology. II. Spatial models and biomedical applications.* Interdisciplinary applied mathematics. Springer, New York, 2004.

[9] K. M. Page, P. K. Maini, and N. A. M. Monk. Pattern formation in spatially heterogeneous Turing reaction–diffusion models. *Physica D: Nonlinear Phenomena*, 181(1-2):80–101, 2003.

[10] K. M. Page, P. K. Maini, and N. A. M. Monk. Complex pattern formation in reaction–diffusion systems with spatially varying parameters. *Physica D: Nonlinear Phenomena*, 202(1-2):95–115, 2005.

[11] Thomas E Woolley, Andrew L Krause, and Eamonn A Gaffney. Bespoke Turing systems. *Bulletin of Mathematical Biology*, 83(5):1–32, 2021.

[12] Zunyi Zhang, Yu Lan, Yang Chai, and Rulang Jiang. Antagonistic actions of msx1 and osr2 pattern mammalian teeth into a single row. *Science*, 323(5918):1232–1234, 2009.
