# Supplementary material for: Bat teeth illuminate the diversification of mammalian tooth classes
Source: Nat Commun. 2023 Aug 22;14:4687. doi: 10.1038/s41467-023-40158-4 (PMC10444822; doi:10.1038/s41467-023-40158-4)
Supplement: Supplementary file 3 — Reporting Summary [file 41467_2023_40158_MOESM3_ESM.pdf]

nature portfolio

Corresponding author(s): Alexa Sadier and Karen Sears

Last updated by author(s): Jun 14, 2023

Reporting Summary

Nature Portfolio wishes to improve the reproducibility of the work that we publish. This form provides structure for consistency and transparency in reporting. For further information on Nature Portfolio policies, see our Editorial Policies and the Editorial Policy Checklist.

Statistics

For all statistical analyses, confirm that the following items are present in the figure legend, table legend, main text, or Methods section.

n/a	Confirmed	
☐	☒	The exact sample size (*n*) for each experimental group/condition, given as a discrete number and unit of measurement
☐	☒	A statement on whether measurements were taken from distinct samples or whether the same sample was measured repeatedly
☐	☒	The statistical test(s) used AND whether they are one- or two-sided<br>*Only common tests should be described solely by name; describe more complex techniques in the Methods section.*
☐	☒	A description of all covariates tested
☐	☒	A description of any assumptions or corrections, such as tests of normality and adjustment for multiple comparisons
☒	☐	A full description of the statistical parameters including central tendency (e.g. means) or other basic estimates (e.g. regression coefficient) AND variation (e.g. standard deviation) or associated estimates of uncertainty (e.g. confidence intervals)
☐	☒	For null hypothesis testing, the test statistic (e.g. *F*, *t*, *r*) with confidence intervals, effect sizes, degrees of freedom and *P* value noted<br>*Give P values as exact values whenever suitable.*
☒	☐	For Bayesian analysis, information on the choice of priors and Markov chain Monte Carlo settings
☒	☐	For hierarchical and complex designs, identification of the appropriate level for tests and full reporting of outcomes
☒	☐	Estimates of effect sizes (e.g. Cohen's *d*, Pearson's *r*), indicating how they were calculated

Our web collection on statistics for biologists contains articles on many of the points above.

Software and code

Policy information about availability of computer code

Data collection	Leica TCS-SP8-MP Deep In Vivo Imaging System using LASX software (whole mount embryonic jaw), Leica Confocal SP8-STED/FLIM/FCS using LASX software (slides) - LAS X image processing software (Leica Microsystems Inc., Wetzlar, Germany) - https://www.leica-microsystems.com/products/microscope-software/p/leica-las-x-ls/
Data analysis	Fiji version 1.53t (Image J) for EdU cell counting and adult jaw and teeth measurement<br>RStudio 2022.07.2 Build 576 using R software R version 4.2.2 (2022-10-31 ucrt) for statistical analysis<br>Data files for the simulations of Supplementary Note 1 are available at: DOI: 10.5281/zenodo.8058070

For manuscripts utilizing custom algorithms or software that are central to the research but not yet described in published literature, software must be made available to editors and reviewers. We strongly encourage code deposition in a community repository (e.g. GitHub). See the Nature Portfolio guidelines for submitting code & software for further information.

Data

Policy information about availability of data

All manuscripts must include a data availability statement. This statement should provide the following information, where applicable:

- Accession codes, unique identifiers, or web links for publicly available datasets
- A description of any restrictions on data availability
- For clinical datasets or third party data, please ensure that the statement adheres to our policy

The µCT scans datasets generated during and/or analyzed during the current study are available from the corresponding author on reasonable request as other parts of these scans (incisors, jaw bones, canines) are currently analyzed for another study.
Teeth were measures on museum specimens or using the Animal Diversity Web website for each species mentioned.
All other measurement datasets are included in this published article (and its supplementary information files).
Data simulation files for Supplementary Note 1 are available at: DOI: 10.5281/zenodo.8058070

Human research participants

Policy information about studies involving human research participants and Sex and Gender in Research.

Reporting on sex and gender	N/A
Population characteristics	N/A
Recruitment	N/A
Ethics oversight	N/A

Note that full information on the approval of the study protocol must also be provided in the manuscript.

Field-specific reporting

Please select the one below that is the best fit for your research. If you are not sure, read the appropriate sections before making your selection.

☒ Life sciences ☐ Behavioural & social sciences ☐ Ecological, evolutionary & environmental sciences

For a reference copy of the document with all sections, see nature.com/documents/nr-reporting-summary-flat.pdf

Life sciences study design

All studies must disclose on these points even when the disclosure is negative.

Sample size	For each adult species, two or three specimen were measured depending on museum specimen availability. For each developmental specimen, one or two specimens caught field specimen were used. This limited sampled size is due to the limited availability of fresh caught specimens (wild caught pregnant females) whose number is limited by our permits. However, to overcome this limitation, we took the advantage of closely related species (that exhibit the same phenotype) to straighten our analyses.
Data exclusions	Two bat groups (2P1M/2P2M and 3P2M) were excluded from the analysis (see main text and methods for more details) as the number of species and specimens in these groups were too low - see Methods Bat groups.
Replication	For adult and developmental specimens, each individual was measure three times. For EdU and IHC experiment, each assay was replicated three times on at least two replicates. All statistical analysis and simulations have been run at least three times for accuracy.
Randomization	Samples were place into morphogroups regarding their tooth number and jaw length. These groups are qualitative and representative of the phylogeny and are not necessary equal in size.
Blinding	Blinding was not relevant to this study as measure were taken on skull and teeth without preconception of the data. Regarding experiments (EdU and IHC), images were taken using field numbers with no indication of the species which was unveiled only after the analysis.

Reporting for specific materials, systems and methods

We require information from authors about some types of materials, experimental systems and methods used in many studies. Here, indicate whether each material, system or method listed is relevant to your study. If you are not sure if a list item applies to your research, read the appropriate section before selecting a response.

Materials & experimental systems

n/a	Involved in the study
☐	☒ Antibodies
☒	☐ Eukaryotic cell lines
☒	☐ Palaeontology and archaeology
☐	☒ Animals and other organisms
☒	☐ Clinical data
☒	☐ Dual use research of concern

Methods

n/a	Involved in the study
☒	☐ ChIP-seq
☒	☐ Flow cytometry
☒	☐ MRI-based neuroimaging

Antibodies

Antibodies used	IHC experiments: Primary: PCNA antibody Rabbit mAB #13110 (Lot 7) Cell Signaling technology, Secondary: HCR-IHC kit using Donkey Anti-Rabbit Ab-B2 (Molecular Instrument).<br>EdU experiment: Click-iT EdU Alexa fluor 647 labelling kit.
Validation	Signal was observed in proliferating regions for both IHC and EdU experiments.

Animals and other research organisms

Policy information about studies involving animals; ARRIVE guidelines recommended for reporting animal research, and Sex and Gender in Research

Laboratory animals	No laboratory animal were used for this study.
Wild animals	Field specimens were collected in the field (see Supplementary Table 1) using mist nets, harp traps or butterfly nets. Pregnant females were staged by palpation and euthanized humanely the night of their capture with isoflurane according to approved institutional animal care and use committee (IACUC) protocols 14199 at UIUC, 2017-093 at UCLA and the following permits Dominican Republic: VAPB-01436; Puerto Rico: 2015-EPE-028; Trinidad: 000619 and 000620 Apr 18th 2018. Bats were kept in cloth bags per standard procedures prior being euthanized for embryo and tissue collection (no more than a few hour in the same night). Lethal sampling is the only way for collecting embryos of different stages in mammals. The number of lethal sampling in reduced to the minimum according to our IACUC protocols and other parts of the bats (various tissues from various organs) are kept for other studies and collaboration. Released bats (>95% were release on their site of capture after been given sugar water or fruit).<br><br>Euthanized pecimens were then fixed ON at 4C with PFA and dehydrated the next day in 100% methanol and stored at -20C until used.<br>Species used: Artibeus jamaicensis, Artibeus phaeotis, Carollia perspicillata, Erophylla sezekorni, Glossophaga soricina, Monophyllus redmani, Mormoops blainvillei, Pteronotus quadridens, Uroderma bilobatum. Animals were adults pregnant females whose age can not be determined precisely in the field.
Reporting on sex	For adult museum specimen, sex was balanced when possible depending in the availability (See Supplementary Data 1 for the specimen list), bat exhibit a limited tooth variability between sexes (Munoz-Romo et al. 2021) and the potential differences dues to body size were normalized by body mass. Embryos were collected from pregnant female but sex was not assessed (which is not always possible for early stages).
Field-collected samples	Field specimens were collected in the field (see Supplementary Data 1) using mist nets, harp traps or butterfly nets and euthanized humanely with isoflurane after capture according to approved institutional animal care and use committee (IACUC) protocols 14199 at UIUC, 2017-093 at UCLA and the following permits Dominican Republic: VAPB-01436; Puerto Rico: 2015-EPE-028; Trinidad: 000619 and 000620 Apr 18th 2018. Specimens were then fixed ON at 4C with PFA and dehydrated the next day in 100% methanol and stored at -20C until used. Bats were kept in cloth bags that were hanged on a line between trees between capture and lethal termination on site. Bats were not housed or transported on long distances.
Ethics oversight	Ethical approval has been performed by the institutional animal care and use committee (IACUC) protocols 14199 at UIUC, 2017-093 at UCLA. Institutional animal care and use committee (IACUC) protocols 14199 at UIUC, 2017-093 at UCLA and the following permits Dominican Republic: VAPB-01436; Puerto Rico: 2015-EPE-028; Trinidad: 000619 and 000620 Apr 18th 2018.

Note that full information on the approval of the study protocol must also be provided in the manuscript.
