# Supplementary material for: Bat teeth illuminate the diversification of mammalian tooth classes
Source: Nat Commun. 2023 Aug 22;14:4687. doi: 10.1038/s41467-023-40158-4 (PMC10444822; doi:10.1038/s41467-023-40158-4)
Supplement: Supplementary file 4 — Description of Additional Supplementary Files [file 41467_2023_40158_MOESM4_ESM.pdf]

Description of Additional Supplementary Files

Supplementary Data 1
Description: Tab “Adult specimens”; adult specimens used for adult measurements and their accession numbers (museum specimens) or field number (field caught specimens). Tab “Embryos”; embryonic specimens used for developmental experiments with their Field number (Specimen number) and Field site (location caught).

Supplementary Data 2
Description: Body mass, dental formula and diet for each bat species in this study.

Supplementary Data 3
Description: ANOVA-Tukey HSD test (single-step multiple comparisons of means, one-sided) of each morphogroup comparing tooth sizes.
